# Early Identification of Traumatic Durotomy Associated with Atlantooccipital Dislocation May Prevent Retropharyngeal Pseudomeningocele Development

**DOI:** 10.1155/2015/361764

**Published:** 2015-04-30

**Authors:** Robert S. Qiu, Mina G. Safain, Max Shutran, Alejandra M. Hernandez, Steven W. Hwang, Ron I. Riesenburger

**Affiliations:** Department of Neurosurgery, Tufts Medical Center and Tufts University School of Medicine, Boston, MA 02111, USA

## Abstract

Atlantooccipital dislocation can be complicated by a traumatic durotomy that may lead to the rare development of a retropharyngeal pseudomeningocele. To our knowledge this has been reported only five times previously. We present the case of a 60-year-old man involved in a motor vehicle accident who suffered an atlantooccipital dislocation and C5-C6 three-column injury. A unique MRI image of a defect in the ventral dura posterior to C2 was appreciated. He underwent occiput to T2 internal fixation and arthrodesis. During surgery, CSF egress was seen caudal to the right C2 nerve root. A DuraMatrix onlay patch reinforced with DuraSeal was placed to stop the CSF leak. A lumbar subarachnoid drain was also placed. The patient made a satisfactory recovery with residual mild weakness of his right upper extremity. In this report, we demonstrate that careful MRI review can reveal a ventral durotomy in a traumatic atlantooccipital dislocation and, if discovered, effective treatment including a lumbar subarachnoid drain for CSF diversion may prevent progression to a retropharyngeal pseudomeningocele. The literature on this rare presentation and associated durotomy is provided.

## 1. Introduction

Traumatic atlantooccipital dislocation (AOD) is an uncommon injury that frequently results in death. Improvements in care and early diagnosis of the condition have led to an increased number of patients surviving, but this disease entity is still associated with significant morbidity and complications [1–3]. An unusual complication associated with AOD is the development of a retropharyngeal pseudomeningocele in a delayed fashion with five current reports in the literature [2–6]. Its development is the result of cerebrospinal fluid extravasating and collecting in the retropharyngeal space in the setting of a traumatic durotomy [2, 4, 6]. Previous reports have suggested that a delay in the diagnosis of a retropharyngeal pseudomeningocele can lead to worse outcomes and can be associated with development of hydrocephalus and syringomyelia [2–6].

**Figure 1:**
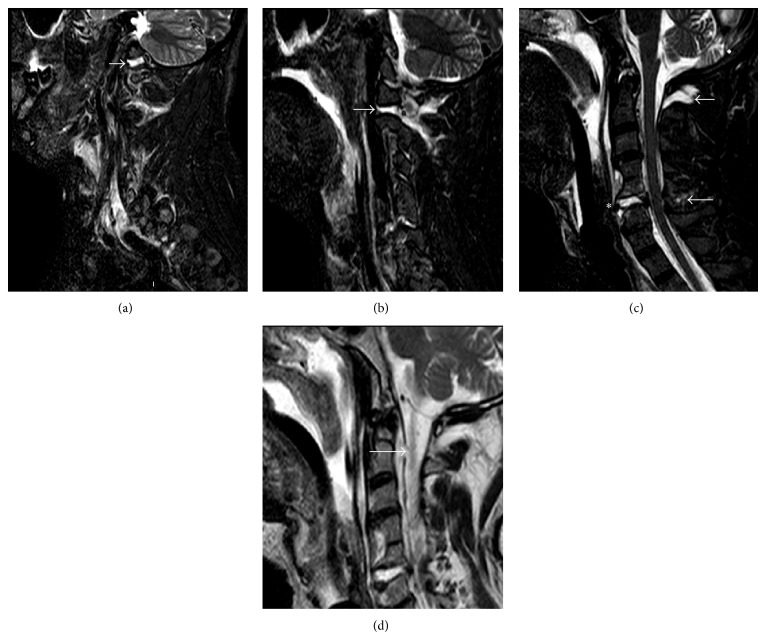
Multiple preoperative sagittal magnetic resonance images (MRI). (a) Left parasagittal short tau inversion recovery (STIR) image demonstrating abnormal fluid in the left atlantooccipital joint (arrow). (b) Left parasagittal STIR image demonstrating abnormal fluid in the left C1-C2 joint (arrow). (c) Midline sagittal STIR image demonstrating disk disruption at the C5-C6 level (∗) and abnormal signal demonstrating posterior ligamentous injury at the occiput-C1 level (rostral arrow) and at the C5-C6 level (caudal arrow). (d) Sagittal T2-weighted MRI demonstrating ventral dural defect at the level of C2 (arrow).

Most reported cases in the literature have intraoperatively identified the presence of a durotomy after the development of a symptomatic retropharyngeal pseudomeningocele. In the current report, we present a case of AOD where a traumatic durotomy was seen on magnetic resonance imaging (MRI) upon initial patient presentation. This unique image of the ventral dural tear was captured and is presented here (Figure 1). The patient had posterior surgery to stabilize the AOD, and cerebrospinal fluid (CSF) extravasation was treated with a dural patch and 5 days of cerebrospinal fluid diversion via lumbar subarachnoid drainage. The patient did not develop a symptomatic retropharyngeal pseudomeningocele and has done well neurologically.

## 2. Case Report

A 60-year-old unrestrained intoxicated driver presented to our emergency department after a motor vehicle accident. Initial examination revealed proximal upper extremity weakness and severe hoarseness, concerning for vagus nerve injury. He was intubated for airway protection and had subsequent computed tomography (CT) and MRI of his cervical spine. MRI demonstrated evidence of AOD (Figure 1). Short tau inversion recovery (STIR) images demonstrated a large amount of fluid in both atlantooccipital joints (Figure 1(a)) as well as both C1-C2 joint complexes (Figure 1(b)). A three-column C5-C6 injury was also apparent and involved the disk space and posterior ligamentous complex (Figure 1(c)). Importantly, the MRI demonstrated a large defect in the ventral dura at the C2 level (Figure 1(d)).

**Figure 2:**
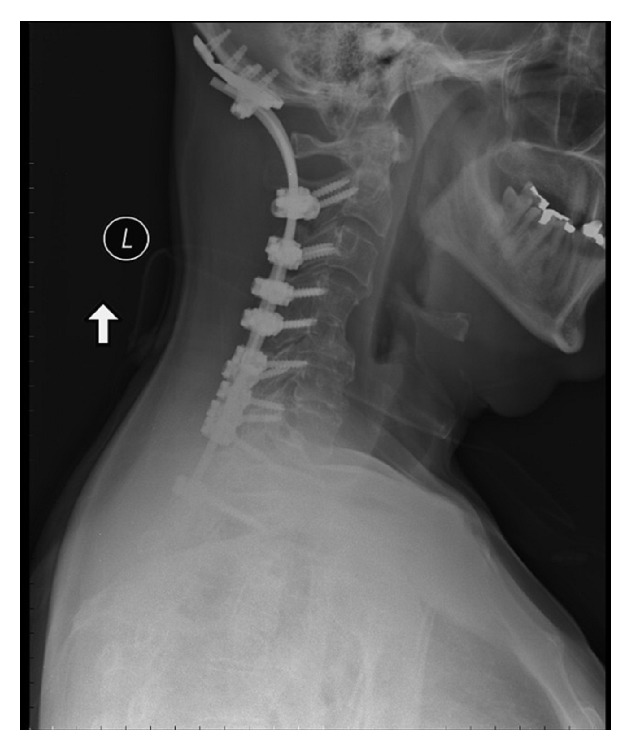
Postoperative lateral X-ray demonstrating posterior instrumentation from the occiput to T2.

The patient was thought to have two unstable cervical spine injuries and was taken to the operating room for immediate surgical stabilization. He underwent a posterior internal fixation and arthrodesis from occiput to T2 (Figure 2). During surgery, a CSF leak was encountered emanating caudal to the right C2 nerve root. The origin of the CSF leak was unable to be visualized due to its ventral location. To prevent posterior CSF egress, a DuraMatrix patch (Stryker, MI, USA) supplemented with DuraSeal (Covidien, MA, USA) was placed caudal to the C2 nerve root. While we felt this technique may prevent CSF egress posteriorly, thus preventing a potential postoperative cerebrospinal-cutaneous fistula, it did not address the defect in the ventral dura. For this reason, a lumbar subarachnoid drain was placed for CSF diversion with the goal that the ventral durotomy would scar. CSF drainage was continued at 10 mL per hour for 5 days. The patient was extubated 4 days after injury with continued hoarseness after extubation. A percutaneous gastrostomy tube was placed for feeding and removed at 2 months after his hoarseness resolved. One-year follow-up demonstrates some slight dysphagia, full strength in his left upper extremity, and mild weakness (4/5) in his right upper extremity.

## 3. Discussion

Traumatic AOD can involve a ventral durotomy that has been reported to develop into a retropharyngeal pseudomeningocele. However, the exact incidence of retropharyngeal pseudomeningocele development in AOD is unknown because there are only a few reports in the literature (Table 1) [2–6]. In our analysis of those case series with 3 or more patients with AOD, the incidence of traumatic durotomy varied from 6% to 75% [7, 8] but was not mentioned in the vast majority of case series [9–14]. Thus, it is currently uncertain what percentage of patients with AOD will have a traumatic durotomy and what percentage will subsequently develop retropharyngeal pseudomeningocele if left untreated.

Currently all reported retropharyngeal pseudomeningoceles were not diagnosed until several weeks to several months later, usually following symptoms of dysphagia and/or respiratory distress [2–6]. However, these presenting symptoms are not specific because they can also be attributed to bulbar-medullary and vagal nerve injury sustained during the AOD [1–4]. Cases of retropharyngeal pseudomeningocele have also been associated with hydrocephalus and syringomyelia, both resulting in worse outcomes [3, 5, 6]. In patients with delayed dysphagia, MRI of the brain and cervical spine is indicated to look for a retropharyngeal pseudomeningocele and concurrently rule out syringomyelia and hydrocephalus. In our case, preoperative MRI clearly identified a traumatic durotomy which was corroborated by intraoperative findings and modified our management (Figure 1(d)).

Initial conservative treatment of a retropharyngeal pseudomeningocele with head elevation, bed rest, and acetazolamide was not successful as reported by Cognetti et al. [2] and these authors advocated surgical placement of cerebrospinal diverting shunts. Resolution of retropharyngeal pseudomeningoceles has been accomplished with ventriculoperitoneal shunting in patients with concomitant hydrocephalus [3, 5] and lumboperitoneal shunting in patients with pseudomeningocele without associated hydrocephalus [2, 3].

In this current report, a ventral traumatic durotomy was seen on MRI and CSF egress was confirmed during surgery. Because of the morbidity associated with the development of a retropharyngeal pseudomeningocele, MRI scans that demonstrate AOD should be assessed for a ventral durotomy. Early identification of this abnormality can suggest to the surgeon that additional treatments during surgical stabilization may be necessary in order to prevent formation of a retropharyngeal pseudomeningocele. In this case, treatment of a ventral dural tear was not completely accomplished with a dural substitute patch. While this step helps prevent posterior cerebrospinal fluid egress and decrease the likelihood of developing a posterior CSF-cutaneous fistula, it does not treat the ventral durotomy. We felt that the ventral durotomy should be addressed to decrease the chance of retropharyngeal pseudomeningocele development. However, we also felt that an additional transoral surgical approach for direct primary repair was not indicated because it would carry a high risk of postoperative meningitis. We therefore elected to treat this with the addition of a lumbar subarachnoid drain for aggressive cerebrospinal fluid diversion over five days. We thought that this would likely give the ventral durotomy enough time to seal.

We theorize that, in order for a retropharyngeal pseudomeningocele to form, several atlantooccipital structures must be injured. We suspect this would include a ventral durotomy, a disruption of the PLL/tectorial membrane, and ALL/anterior atlantooccipital membrane. It is difficult to clearly identify complete disruptions to the PLL/tectorial membrane and ALL/anterior atlantooccipital membrane on MRI imaging. However, this study shows that careful MRI review can reveal a ventral durotomy following AOD. In this situation, we recommend strong consideration for placement of a lumbar subarachnoid drain as a temporary measure that may prevent retropharyngeal pseudomeningocele development and avoid the need for permanent CSF diversion. We, like other authors, recommend repeat imaging to rule out a retropharyngeal pseudomeningocele should a patient develop a delayed inability to swallow [5, 6].

## Figures and Tables

**Table 1:** Cases of retropharyngeal pseudomeningocele following AOD.

Author	Year	Age	AOD management	Symptom	Diagnosis of RP	Hydrocephalus	Treatment of RP	Outcome
Williams et al. [3]	1995	3.5	Arthrodesis	Respiratory and dysphagia	4 weeks	Yes	VP shunt	Resolution of RP
Naso et al. [5]	1997	26	Halo	Respiratory and dysphagia	3.5 months	Yes	VP shunt	Resolution of RP
	1997	11	Unknown	Respiratory	5 weeks	Yes	None	Died
Reed et al. [6]	2005	9	Arthrodesis	Incidental	4 weeks	Yes	None	Died
Cognetti et al. [2]	2006	19	Arthrodesis	Dysphagia	6 weeks	No	LP shunt	Resolution of RP
Gutiérrez-González et al. [4]	2008	29	Hard collar	Respiratory	3 weeks	No	None	Died
Qiu et al. (current report)	2013	60	Arthrodesis	Respiratory, weakness	No RP	No	None	No RP

AOD: atlantooccipital dislocation, VP: ventriculoperitoneal, LP: lumboperitoneal, and RP: retropharyngeal pseudomeningocele.

## References

[1] (2002). Diagnosis and management of traumatic atlanto-occipital dislocation injuries. *Neurosurgery*.

[2] Cognetti D. M., Enochs W. S., Willcox T. O. (2006). Retropharyngeal pseudomeningocele presenting as dysphagia after atlantooccipital dislocation. *Laryngoscope*.

[3] Williams M. J., Elliott J. L., Nichols J. (1995). Atlantooccipital dislocation: a case report. *Journal of Clinical Anesthesia*.

[4] Gutiérrez-González R., Boto G. R., Pérez-Zamarrón Á., Rivero-Garvía M. (2008). Retropharyngeal pseudomeningocele formation as a traumatic atlanto-occipital dislocation complication: case report and review. *European Spine Journal*.

[5] Naso W. B., Cure J., Cuddy B. G. (1997). Retropharyngeal pseudomeningocele after atlanto-occipital dislocation: report of two cases. *Neurosurgery*.

[6] Reed C. M., Campbell S. E., Beall D. P., Bui J. S., Stefko R. M. (2005). Atlanto-occipital dislocation with traumatic pseudomeningocele formation and post-traumatic syringomyelia. *Spine.*.

[7] Adams V. I. (1992). Neck injuries: I. Occipitoatlantal dislocation—a pathologic study of twelve traffic fatalities. *Journal of Forensic Sciences*.

[8] Bellabarba C., Mirza S. K., West G. A. (2006). Diagnosis and treatment of craniocervical dislocation in a series of 17 consecutive survivors during an 8-year period. *Journal of Neurosurgery: Spine*.

[9] Grabb B. C., Frye T. A., Hedlund G. L., Vaid Y. N., Grabb P. A., Royal S. A. (1999). MRI diagnosis of suspected atlanto-occipital dissociation in childhood. *Pediatric Radiology*.

[10] Horn E. M., Feiz-Erfan I., Lekovic G. P., Dickman C. A., Sonntag V. K. H., Theodore N. (2007). Survivors of occipitoatlantal dislocation injuries: Imaging and clinical correlates. *Journal of Neurosurgery: Spine*.

[11] Matava M. J., Whitesides T. E., Davis P. C. (1993). Traumatic atlanto-occipital dislocation with survival: Serial computerized tomography as an aid to diagnosis and reduction: a report of three cases. *Spine*.

[12] Maves C. K., Souza A., Prenger E. C., Kirks D. R. (1991). Traumatic atlanto-occipital disruption in children. *Pediatric Radiology*.

[13] Montane I., Eismont F. J., Green B. A. (1991). Traumatic occipitoatlantal dislocation. *Spine*.

[14] Pang D., Nemzek W. R., Zovickian J. (2007). Atlanto-occipital dislocation—part 2: the clinical use of (occipital) condyle-C1 interval, comparison with other diagnostic methods, and the manifestation, management, and outcome of atlanto-occipital dislocation in children. *Neurosurgery*.

